# Trends in Risks for Second Primary Cancers Associated With Index Human Papillomavirus–Associated Cancers

**DOI:** 10.1001/jamanetworkopen.2018.1999

**Published:** 2018-09-07

**Authors:** Ryan Suk, Parag Mahale, Kalyani Sonawane, Andrew G. Sikora, Jagpreet Chhatwal, Kathleen M. Schmeler, Keith Sigel, Scott B. Cantor, Elizabeth Y. Chiao, Ashish A. Deshmukh

**Affiliations:** 1 Department of Management Policy and Community Health, The University of Texas Health Science Center at Houston, School of Public Health, Houston; 2 Division of Cancer Epidemiology and Genetics, National Cancer Institute, Bethesda, Maryland; 3 Department of Otolaryngology–Head and Neck Surgery, Baylor College of Medicine, Houston, Texas; 4 Massachusetts General Hospital Institute for Technology Assessment, Harvard Medical School, Boston; 5 Department of Gynecologic Oncology and Reproductive Medicine, The University of Texas MD Anderson Cancer Center, Houston; 6 Division of General Internal Medicine, Department of Medicine, Icahn School of Medicine at Mount Sinai, New York, New York; 7 Department of Health Services Research, The University of Texas MD Anderson Cancer Center, Houston; 8 Section of Infectious Diseases, Department of Medicine, Baylor College of Medicine, Houston, Texas

## Abstract

**Importance:**

In the last 4 decades, survival among patients with human papillomavirus (HPV)–associated cancers has improved, while the incidence of these cancers has increased among younger cohorts. Among survivors of HPV-associated cancers, persistent HPV infection may remain a risk factor for preventable HPV-associated second primary cancers (HPV-SPCs).

**Objectives:**

To investigate the risk of HPV-SPCs among survivors of HPV-associated index cancers and to test the hypothesis that the HPV-SPC risk among these persons has increased over the last 4 decades.

**Design, Setting, and Participants:**

A retrospective cohort study of 9 cancer registries of the Surveillance, Epidemiology, and End Results (SEER) database was conducted to identify patients with HPV-associated (cervical, vaginal, vulvar, oropharyngeal, anal, and penile) cancers diagnosed from January 1, 1973, through December 31, 2014. The dates of analysis were July 1, 2017, to January 31, 2018.

**Main Outcomes and Measures:**

The HPV-SPC risk was quantified by calculating standard incidence ratios (SIRs) and excess absolute risks (EARs) per 10 000 person-years at risk (PYR). The HPV-SPC risk by time was estimated using Poisson regression.

**Results:**

From 113 272 (73 085 female and 40 187 male) survivors of HPV-associated cancers, 1397 women and 1098 men developed HPV-SPCs. The SIRs for HPV-SPCs were 6.2 (95% CI, 5.9-6.6) among women and 15.8 (95% CI, 14.9-16.8) among men. The EARs were 18.2 per 10 000 PYR for women and 53.5 per 10 000 PYR for men. Among both women and men, those who had index oropharyngeal cancers had the highest HPV-SPC risk (SIR, 19.8 [95% CI, 18.4-21.4] and EAR, 80.6 per 10 000 PYR among women; SIR, 18.0 [95% CI, 16.9-19.1] and EAR, 61.5 per 10 000 PYR among men). Women who had index cervical cancers and men who had index anal cancers had the lowest HPV-SPC risk (SIR, 2.4 [95% CI, 2.2-2.7] and EAR, 4.5 per 10 000 PYR among women; SIR, 6.5 [95% CI, 4.7-8.8] and EAR, 18.5 per 10 000 PYR among men). Both women and men who had index HPV-associated cancers of any kind had a significantly higher risk of oropharyngeal HPV-SPCs. Over the last 4 decades, the risk of developing most types of HPV-SPCs after index cervical, vaginal, and vulvar cancers increased.

**Conclusions and Relevance:**

According to this study, the HPV-SPC risk among survivors of HPV-associated cancers is significant, implying that persistent HPV infection at multiple sites may be associated with HPV-SPCs. These findings have the potential to inform surveillance recommendations for survivors of HPV-associated cancers.

## Key Points

**Question:**

What is the risk of second primary human papillomavirus (HPV)–associated cancer among survivors of HPV-associated cancers?

**Finding:**

In this cohort study of 113 272 survivors of index HPV-associated cancers, the incidence of most types of second primary HPV-associated cancers (vaginal, vulvar, oropharyngeal, anal, and penile cancers) was high and has increased over the last 4 decades.

**Meaning:**

Persistent HPV infection at multiple sites might lead to second primary HPV-associated cancers, suggesting the need for increased screening for the detection of HPV-associated precancerous and early cancerous lesions among survivors of HPV-associated cancers.

## Teaser

This registry-based cohort study using data from the Surveillance, Epidemiology, and End Results (SEER) database investigates the risk of human papillomavirus (HPV)–associated second primary cancers among survivors of HPV-associated index cancers and tests the hypothesis that the risk of HPV-associated second primary cancer has increased over the last 4 decades.

## Introduction

Human papillomavirus (HPV) causes one of the largest families of cancers, including gynecological (cervical, vaginal, and vulvar), anogenital (anal and penile), and oropharyngeal cancers.^1^ More than 150 000 (41 000 per year) new cases of HPV-associated cancers were diagnosed between 2010 and 2014 in the United States.^2^ Cervical cancer is the most common HPV-associated cancer among women (11 670 cases per year), and oropharyngeal cancers are the most common among men (13 976 cases per year).^2^

Substantial advances in cancer diagnosis and management have been made in the last few decades, leading to steady improvements in the survival rates of several patient populations with cancer. With advances in treatment technology that have led to longer survival durations among an aging, at-risk population,^3,4,5^ the population of cancer survivors is rapidly growing. As of January 2016, there were more than 15 million cancer survivors in the United States, representing almost 5% of the US population, and this number is projected by 2020 to increase to 18 million.^6^ Because of increased survivorship, the population at risk of developing second primary cancers (SPCs) has also increased,^7^ making SPCs the leading causes of morbidity, mortality, and economic burden among cancer survivors.^8,9,10,11^

Most HPV-associated cancers are preventable if precancerous lesions are detected and adequately managed. Even among survivors of HPV-associated cancers who develop invasive HPV-associated SPCs (HPV-SPCs), early detection can provide a survival benefit.^12,13,14,15^ However, with the exception of cervical carcinoma, screening recommendations for survivors of most types of HPV-associated cancers are lacking, potentially putting individuals at risk of developing aggressive, difficult-to-treat HPV-SPCs before the disease is diagnosed. Studying the association between HPV-associated index primary cancers and HPV-SPCs occurring at various anatomic sites could (1) inform and guide surveillance recommendations for and the clinical management of survivors of such cancers, (2) highlight the need for improvement in screening technology for the detection of specific HPV-associated precancerous or early cancerous lesions among survivors of HPV-associated cancers, and (3) help us understand interactions between HPV infections occurring at various anatomic sites and their role in HPV-associated carcinogenesis.

In the present study, our first objective was to investigate the HPV-SPC risk among survivors of index HPV-associated cancers substratified by cancer site. Our second objective was to characterize the HPV-SPC risk by calendar year, testing the hypothesis that the HPV-SPC risk among survivors of HPV-associated cancers is increasing.

## Methods

### Surveillance, Epidemiology, and End Results Program and Patient Identification

We performed a retrospective cohort study using data from the Surveillance, Epidemiology, and End Results (SEER) Program database. The database includes 18 cancer registries, which represent approximately 28% of the US population. Individuals with cancer in the SEER database are also linked to any subsequent incident cancer diagnoses. Our analysis included data from 9 registries—Atlanta (Georgia), Connecticut, Detroit (Michigan), Hawaii, Iowa, New Mexico, San Francisco–Oakland (California), Seattle–Puget Sound (Washington), and Utah—that have been contributing to the SEER data since 1973, which enabled us to assess trends in cancer incidence over the years.^16^ The present study was conducted in compliance with the National Cancer Institute’s SEER limited-use data agreement.

We identified survivors of HPV-associated cancers diagnosed from January 1, 1973, through December 31, 2014. We used the Centers for Disease Control and Prevention criteria to identify HPV-associated cancers,^2,17,18,19,20^ which were defined as invasive cancers located at specific anatomic sites and comprising specific cell types in which HPV DNA is frequently found; these cancers comprised squamous cell carcinomas (SCCs) of the vagina, vulva, oropharynx, anus, and penis and all carcinomas of the cervix, including adenocarcinomas and SCCs. Oropharyngeal cancers included cancers of the base of the tongue; pharyngeal tonsils, anterior and posterior tonsillar pillars, and glossotonsillar sulci; anterior surface of the soft palate and uvula; and lateral and posterior pharyngeal walls. All cancers were malignant, classified by anatomic site using the *International Classification of Diseases for Oncology*, *Third Edition* (*ICD-O-3*), and confirmed histologically. Detailed *ICD-O-3* site codes, histology codes, and additional restrictions that were used to define index cancers and SPCs are listed in eTable 1 in the Supplement. We excluded persons with unknown age or those whose cancer was diagnosed at autopsy or was first documented on the death certificate (<1% of total patients for each cancer).

The analysis followed the Strengthening the Reporting of Observational Studies in Epidemiology (STROBE) guidelines.^21^ The dates of analysis were July 1, 2017, to January 31, 2018, and the University of Florida institutional review board approved this study as exempt.

### HPV-SPC Definition and Study Outcomes

An HPV-SPC was defined as a metachronous invasive solid cancer developing at least 2 months after the diagnosis of index HPV-associated cancers using the criteria by Warren and Gates^22^ as modified by the National Cancer Institute.^23^ The first 2 months were excluded because the intensified screening of patients with cancer during the initial 2 months after cancer diagnosis may lead to the identification of synchronous cancers. The primary outcome of interest was the HPV-SPC risk among survivors of HPV-associated cancers.

### Statistical Analysis

Participants were followed up starting 2 months after the diagnosis of an index primary cancer until HPV-SPC diagnosis, death, last follow-up, or the end of the study period (December 31, 2014), whichever occurred first. To investigate the HPV-SPC risk, we used standardized incidence ratios (SIRs), which were originally described by Schoenberg and Myers^24^ and adapted to cancer registry analysis by Begg et al.^25^ The SIRs (a relative measure of the strength of association between 2 cancers) were calculated as the ratio of observed HPV-SPC cases to those expected in the general population. The expected numbers of cases were estimated based on reference rates stratified by race/ethnicity, sex, age interval, and calendar year in the 9 SEER registries. We tested the statistical significance assuming that the observed number of second cancers approximated a Poisson distribution and that no variation was associated with the expected number of cases. We then used Byar approximation to calculate 95% CIs for the SIRs.^23^ The SIRs whose 95% CI excluded 1.0 were considered statistically significant (2-sided *P* < .05). Excess absolute risks (EARs), which represent the absolute number of additional subsequent cancers attributed to index HPV-associated cancers, were calculated as the excess number of second cancers (ie, the number of observed cancers minus the number of expected cancers) among patients with index HPV-associated cancer per 10 000 person-years at risk (PYR). Finally, we investigated the cumulative risk of developing HPV-SPCs using Fine and Gray competing risk proportional hazards models.^26^

We also analyzed the HPV-SPC risk by calendar year and latency interval. Trends in the SIRs by calendar year and latency interval were calculated by fitting Poisson regression models for each cancer type.^27^ All analyses were stratified by sex. The SIRs were calculated using SEER*Stat (version 8.3.2; National Cancer Institute Cancer Statistics Branch). Additional analyses were performed using SAS (version 9.4; SAS Institute Inc).

Because differentiating between cancer recurrences and SPCs at the same site can be difficult, we also performed some sensitivity analyses. First, we evaluated the overall risk of developing HPV-SPCs at sites different from that of the original cancer. Second, in our estimation of the HPV-SPC risk, we included only those HPV-SPCs diagnosed more than 1 year after the diagnosis of the index cancer. Third, to investigate whether the risk of HPV-SPCs is in excess because of HPV infection, we estimated the SIR for HPV-SPC among survivors of non–HPV-associated cancers (ie, prostate, breast, and thyroid).

## Results

The cohort included 113 272 survivors of HPV-associated cancers (73 085 women and 40 187 men) who were followed up for 837 131 person-years (644 691 person-years for women and 192 440 person-years for men). Of these survivors, 5469 (4.8%) were lost to follow-up, leaving 107 803 (69 949 women and 37 854 men) to be included in our analysis (eFigure 1 in the Supplement).

The characteristics of the HPV-associated index primary cancer cohort are summarized in Table 1. The median age at HPV-SPC diagnosis and the median time to HPV-SPC diagnosis differed by the index cancer type and SPC type. For instance, among women, the median age at second anal cancer diagnosis after index cervical or vulvar cancer was 63 and 61 years, respectively, while the median time between cervical and anal cancer diagnoses was 13.8 years and between vulvar and anal cancer diagnoses was 8.3 years. Among men, the median age at second oropharyngeal cancer after index penile cancer was 76 years, and the median time between diagnoses was 4.5 years; the median age at second oropharyngeal cancer after index anal cancer was 57 years, and the median time between diagnoses was 2.7 years.

**Table 1.** Descriptive Characteristics of Cases of Index Human Papillomavirus–Associated Cancers Using Surveillance, Epidemiology, and End Results Program Data From 1973 to 2014

Characteristic	Women					Men		
	Cervical (n = 44 011)	Vaginal (n = 1673)	Vulvar (n = 6905)	OPC (n = 15 303)	Anal (n = 5193)	Penile (n = 2721)	OPC (n = 34 248)	Anal (n = 3218)
Age at diagnosis, y, No. (%)								
<30	3554 (8.1)	12 (0.7)	67 (1.0)	141 (0.9)	11 (0.2)	16 (0.6)	179 (0.5)	32 (1.0)
30-39	9853 (22.4)	53 (3.2)	354 (5.1)	434 (2.8)	141 (2.7)	75 (2.8)	830 (2.4)	265 (8.2)
40-49	10 047 (22.8)	155 (9.3)	789 (11.4)	1427 (9.3)	756 (14.6)	261 (9.6)	4795 (14.0)	710 (22.1)
50-59	7960 (18.1)	258 (15.4)	1068 (15.5)	3481 (22.7)	1335 (25.7)	511 (18.8)	10 929 (31.9)	911 (28.3)
60-69	6345 (14.4)	344 (20.6)	1209 (17.5)	4099 (26.8)	1330 (25.6)	732 (26.9)	10 331 (30.2)	731 (22.7)
70-79	3967 (9.0)	401 (24.0)	1676 (24.3)	3354 (21.9)	971 (18.7)	703 (25.8)	5289 (15.4)	396 (12.3)
≥80	2285 (5.2)	450 (26.9)	1742 (25.2)	2367 (15.5)	649 (12.5)	423 (15.5)	1895 (5.5)	173 (5.4)
Year of diagnosis, No. (%)								
1973-1979	8440 (19.2)	274 (16.4)	840 (12.2)	2098 (13.7)	417 (8.0)	439 (16.1)	3982 (11.6)	230 (7.1)
1980-1989	10 860 (24.7)	421 (25.2)	1368 (19.8)	3589 (23.5)	842 (16.2)	618 (22.7)	6629 (19.4)	422 (13.1)
1990-1999	10 937 (24.9)	364 (21.8)	1729 (25.0)	3518 (23.0)	1052 (20.3)	590 (21.7)	7182 (21.0)	693 (21.5)
2000-2009	9198 (20.9)	394 (23.6)	1887 (27.3)	3842 (25.1)	1699 (32.7)	678 (24.9)	9722 (28.4)	1164 (36.2)
2010-2014	4576 (10.4)	220 (13.2)	1081 (15.7)	2256 (14.7)	1183 (22.8)	396 (14.6)	6733 (19.7)	709 (22.0)
Race/ethnicity, No. (%)								
White	32 903 (74.8)	1335 (79.8)	6126 (88.7)	12 977 (84.8)	4542 (87.5)	2319 (85.2)	28 616 (83.6)	2663 (82.8)
Black	6558 (14.9)	248 (14.8)	547 (7.9)	1339 (8.7)	477 (9.2)	261 (9.6)	3861 (11.3)	464 (14.4)
American Indian/Alaskan Native	521 (1.2)	10 (0.6)	44 (0.6)	61 (0.4)	24 (0.5)	32 (1.2)	152 (0.4)	19 (0.6)
Asian/Pacific Islander	3672 (8.3)	71 (4.2)	151 (2.2)	854 (5.6)	135 (2.6)	92 (3.4)	1471 (4.3)	56 (1.7)
Unknown	357 (0.8)	9 (0.5)	37 (0.5)	72 (0.5)	15 (0.3)	17 (0.6)	148 (0.4)	16 (0.5)
Marital status at diagnosis, No. (%)								
Single (never married)	7466 (17.0)	169 (10.1)	880 (12.7)	1767 (11.5)	670 (12.9)	402 (14.8)	5632 (16.4)	1448 (45.0)
Married or partnered	20 806 (47.3)	575 (34.4)	2529 (36.6)	6429 (42.0)	2257 (43.5)	1611 (59.2)	19 547 (57.1)	1091 (33.9)
Separated, widowed, or divorced	13 324 (30.3)	825 (49.3)	3007 (43.5)	6068 (39.7)	2036 (39.2)	527 (19.4)	7107 (20.8)	496 (15.4)
Unknown	2415 (5.5)	104 (6.2)	489 (7.1)	1039 (6.8)	230 (4.4)	181 (6.7)	1962 (5.7)	183 (5.7)
Age at diagnosis of index cancer, median (range), y	48 (6-104)	70 (21-104)	69 (12-102)	65 (0-107)	62 (23-105)	67 (17-98)	60 (2-103)	56 (19-103)
Age at diagnosis of SPC, median (range), y								
Cervical	50 (21-91)	71 (42-93)	68 (27-92)	62 (40-89)	64 (44-86)	NA	NA	NA
Vaginal	61 (30-90)	79 (51-88)	79 (24-95)	87 (65-94)	62 (51-75)	NA	NA	NA
Vulvar	65 (33-96)	68 (46-87)	74 (34-99)	70 (52-84)	61 (41-87)	NA	NA	NA
OPC	60 (43-85)	NA	68 (21-85)	70 (28-97)	74 (46-82)	76 (53-88)	64 (30-94)	57 (39-73)
Anal	63 (36-85)	95 (95-95)	61 (24-87)	82 (64-88)	61 (43-98)	85 (85-85)	60 (44-87)	57 (36-86)
Penile	NA	NA	NA	NA	NA	66 (51-92)	73 (49-85)	47 (47-47)
Time to SPC diagnosis, median (range), y								
Cervical	7.1 (0.2-31.9)	5.4 (0.4-26.2)	4.4 (0.2-8.1)	2.6 (0.3-36.6)	4.3 (0.5-8.3)	NA	NA	NA
Vaginal	7.2 (0.2-37.6)	7.8 (1.7-21.4)	3.4 (0.8-27.3)	3.3 (0.6-18.6)	9.7 (1.6-15.8)	NA	NA	NA
Vulvar	8.6 (0.2-36.6)	3.5 (0.4-16.8)	5.2 (0.7-27.0)	4.2 (0.7-18.5)	4.6 (0.2-18.8)	NA	NA	NA
OPC	10.3 (0.7-36.1)	NA	6.0 (0.3-18.4)	5.8 (0.2-37.8)	6.0 (1.4-27.1)	4.5 (0.4-18.8)	5.6 (0.2-32.1)	2.7 (0.2-17.3)
Anal	13.8 (1.3-37.4)	6.3 (6.3-6.3)	8.3 (0.8-35.4)	7.7 (4.9-12.0)	2.5 (0.2-20.9)	6.9 (6.9-6.9)	3.6 (0.4-10.6)	5.2 (0.8-24.5)
Penile	NA	NA	NA	NA	NA	4.9 (0.3-26.1)	1.7 (0.5-7.6)	0.9 (0.9-0.9)

Abbreviations: NA, not applicable; OPC, oropharyngeal cancer; SPC, second primary cancer.

### HPV-SPC Risk Among Survivors of HPV-Associated Cancers

Overall, we identified 2495 HPV-SPCs—1397 among women (SIR, 6.2 [95% CI, 5.9-6.6] and EAR, 18.2 per 10 000 PYR) and 1098 among men (SIR, 15.8 [95% CI, 14.9-16.8] and EAR, 53.5 per 10 000 PYR)—when the analyzed follow-up period began 2 months after the index cancer diagnosis (Table 2). The SIRs were unaltered when the follow-up period began 1 year after the index cancer diagnosis, and 2376 HPV-SPCs (1278 among women and 1098 among men) were identified (eTable 2 in the Supplement). When we estimated the risk of HPV-SPCs that occurred at sites different from those of the index cancers, the SIRs for women (485 cases) and men (44 cases) were still significantly elevated at 3.5 (95% CI, 3.2-3.8) and 2.1 (95% CI, 1.5-2.8), respectively, with EARs of 5.4 and 1.2 per 10 000 PYR, respectively. These rates are disproportionately high compared with the risk of developing HPV-SPCs after non–HPV-associated cancers. Among women, the SIRs of developing HPV-SPCs after index breast (1651 cases) or thyroid (106 cases) cancers were 0.8 (95% CI, 0.8-0.8) and 0.6 (95% CI, 0.5-0.8), respectively; among men, the SIRs after index prostate (1351 cases) and thyroid (40 cases) cancers were 0.7 (95% CI, 0.7-0.8) and 0.9 (95% CI, 0.6-1.2), respectively.

**Table 2.** Standard Incidence Ratio of HPV-SPCs After Index HPV-Associated Cancers Stratified by Sex

Index HPV-Associated Cancers	Women						Men					
	All HPV-SPC			All HPV-SPC (Same Site Excluded)			All HPV-SPC			All HPV-SPC (Same Site Excluded)		
	Observed Cancers, No.	SIR (95% CI)	EAR per 10 000 PYR^a^	Observed Cancers, No.	SIR (95% CI)	EAR per 10 000 PYR^a^	Observed Cancers, No.	SIR (95% CI)	EAR per 10 000 PYR^a^	Observed Cancers, No.	SIR (95% CI)	EAR per 10 000 PYR^a^
All	1397	6.2 (5.9-6.6)	18.2	485	3.5 (3.2-3.8)	5.4	1098	15.8 (14.9-16.8)	53.5	44	2.1 (1.5-2.8)	1.2
Cervical	362	2.4 (2.2-2.7)	4.5	290	3.5 (3.1-3.9)	4.4	NA	NA	NA	NA	NA	NA
Vaginal	26	6.2 (4.0-9.0)	24.0	19	4.8 (2.9-7.5)	16.6	NA	NA	NA	NA	NA	NA
Vulvar	265	12.7 (11.2-14.2)	50.5	90	5.4 (4.4-6.7)	15.2	NA	NA	NA	NA	NA	NA
OPC	669	19.8 (18.4-21.4)	80.6	37	1.6 (1.1-2.2)	1.8	1007	18.0 (16.9-19.1)	61.5	15	1.7 (1.0-2.8)	0.4
Anal	75	5.2 (4.1-6.5)	17.5	49	3.8 (2.8-5.1)	10.5	42	6.5 (4.7-8.8)	18.5	14	2.4 (1.3-4.0)	4.3
Penile	NA	NA	NA	NA	NA	NA	49	7.0 (5.2-9.3)	22.7	15	2.4 (1.3-3.9)	4.7

Abbreviations: EAR, excess absolute risk; HPV, human papillomavirus; HPV-SPC, HPV-associated second primary cancer; NA, not applicable; OPC, oropharyngeal cancer; PYR, person-years at risk; SIR, standard incidence ratio.

^a^ Number of observed cancers minus number of expected cancers per 10 000 PYR.

Among women, the HPV-SPC incidence was highest after index oropharyngeal cancers (SIR, 19.8 [95% CI, 18.4-21.4] and EAR, 80.6 per 10 000 PYR) and lowest after index cervical cancers (SIR, 2.4 [95% CI, 2.2-2.7] and EAR, 4.5 per 10 000 PYR) (Table 2). Among men, the HPV-SPC incidence was highest after index oropharyngeal cancers (SIR, 18.0 [95% CI, 16.9-19.1] and EAR, 61.5 per 10 000 PYR) and lowest after index anal cancers (SIR, 6.5 [95% CI, 4.7-8.8] and EAR, 18.5 per 10 000 PYR).

We then evaluated the HPV-SPC incidence by the index cancer type among women (Figure, A and eTable 3 in the Supplement). We found that the risks for HPV-SPCs, except cervical SPCs, were highly increased when the SPCs occurred at the index cancer site. The SIRs for oropharyngeal, vulvar, vaginal, and anal cancers were 57.9, 40.6, 26.1, and 14.6, respectively, whereas that for cervical cancer was 1.1. The HPV-SPC risk differed by index cancer. There was an increased risk of vaginal (SIR, 17.3; 95% CI, 14.3-20.6), vulvar (SIR, 3.8; 95% CI, 3.0-4.7), and anal (SIR, 2.3; 95% CI, 1.6-3.2) SPCs after index cervical cancer; an increased risk of vulvar (SIR, 16.6; 95% CI, 8.8-28.4) and cervical (SIR, 3.4; 95% CI, 1.1-7.9) SPCs after index vaginal cancer; an increased risk of vaginal (SIR, 18.5; 95% CI, 11.8-27.8), anal (SIR, 13.2; 95% CI, 8.9-18.7), and oropharyngeal (SIR, 3.3; 95% CI, 2.1-5.1) SPCs after index vulvar cancer; an increased risk of vulvar (SIR, 9.6; 95% CI, 6.6-13.9), vaginal (SIR, 8.4; 95% CI, 3.4-17.3), and oropharyngeal (SIR, 2.1; 95% CI, 1.0-3.9) SPCs after index anal cancer; and an increased risk of vulvar SPC (SIR, 2.1; 95% CI, 1.1-3.5) after index oropharyngeal cancer. Similarly, among men (Figure, B), the risk of HPV-SPCs occurring at the index cancer site was also elevated. The SIRs for penile, oropharyngeal, and anal cancers were 52.5, 21.0, and 43.0, respectively. The risk of oropharyngeal SPC after index penile cancer (SIR, 2.5; 95% CI, 1.3-4.1) or index anal cancer (SIR, 2.4; 95% CI, 1.3-4.1) was elevated; however, the reverse association (ie, the risk for anal or penile SPC cancer after index oropharyngeal cancer) was not statistically significant. We also show the cumulative subsite-specific risk of developing SPCs over the duration of 30 years in eFigure 2 and eFigure 3 in the Supplement.

**Figure.**
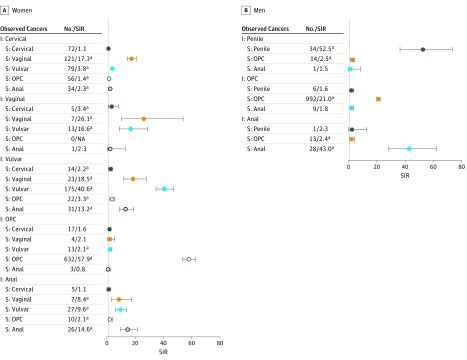
Subsite-Specific Standard Incidence Ratio (SIR) of Human Papillomavirus (HPV)–Associated Second Primary Cancers After HPV-Associated Cancers Shown are subsite-specific SIRs of developing HPV-associated second primary cancers among survivors of index HPV-associated cancers. A, The SIRs of second primary cervical, vaginal, vulvar, oropharyngeal cancer (OPC), and anal cancers after index subsite-specific HPV-associated cancers among women are shown. B, The SIRs of second primary penile, OPC, and anal cancers after index subsite-specific HPV-associated cancers among men are shown. Error bars represent 95% CIs. Each color dot represents a specific HPV-associated second primary cancer. The dotted line indicates an SIR of 1. I indicates index cancer; NA, not applicable; and S, second primary cancer. ^a^Statistically significant at *P* < .05.

We then evaluated the HPV-SPC risk by year of HPV-SPC diagnosis and by latency interval for women (Table 3) and men (Table 4) and report subsite-specific trends. Women’s risk of vaginal SPC after index cervical cancer increased from the 1970s (SIR, 9.2) to the 2010s (SIR, 24.9) (*P* = .04 for trend), whereas their risk of vulvar SPC after index cervical cancer decreased over this same period (SIR, 5.5 and 2.7, respectively; *P* = .03 for trend). Among women, the risk of vulvar SPC at the index cancer site increased from the 1980s (SIR, 7.2) to the 2010s (SIR, 88.7) (*P* < .001 for trend); the risk of oropharyngeal SPC at the index cancer site increased from the 1970s (SIR, 32.5) to the 2010s (SIR, 81.4) (*P* < .001 for trend); and the risk of anal SPC at the index cancer site increased from the 1990s (SIR, 2.9) to the 2010s (SIR, 23.2) (*P* = .004 for trend). As the follow-up interval increased, the risk of vaginal and vulvar SPCs after index cervical cancer, the risk of cervical SPC after index vulvar cancer, and the risk of vulvar SPC after index anal cancer all decreased. Men’s risk of developing anal SPC at the index cancer site increased from the 1990s (SIR, 26.5) to the 2010s (SIR, 74.0) (*P* < .009 for trend), and their risk of penile SPC at the index cancer site increased from the 1980s (SIR, 7.3) to the 2010s (SIR, 180.6) (*P* < .001 for trend).

**Table 3.** Standard Incidence Ratio of HPV-SPCs According to Year of HPV-SPC Diagnosis and Latency Interval for Women

Cancer Site	SIR by Year of HPV-SPC Diagnosis					*P* Value for Trend (Direction of Trend)^a^	SIR by Latency Interval, y				*P* Value for Trend (Direction of Trend)^a^
	1973-1979	1980-1989	1990-1999	2000-2009	2010-2014		<1	1-4	5-9	≥10	
**I: Cervical**											
S: Cervical	NA	0.1^b^	0.8	1.6^b^	2.7^b^	<.001 (+)	2.1^b^	0.9	0.9	1.1	.70
S: Vaginal	9.2^b^	14.9^b^	16.0^b^	16.6^b^	24.9^b^	.04 (+)	27.7^b^	25.2^b^	20.2^b^	12.2^b^	<.001 (−)
S: Vulvar	5.5^b^	4.1^b^	5.7^b^	2.7^b^	2.7^b^	.03 (−)	3.8^b^	4.3^b^	6.6^b^	2.7^b^	.04 (−)
S: OPC	1.8	2.1^b^	1.6	1.2	0.9	.04 (−)	1.4	2.2^b^	0.6	1.4^b^	.39
S: Anal	2.9	3.4^b^	2.8^b^	1.6	2.2^b^	.29	NA	2.8^b^	1.8	2.4^b^	.67
**I: Vaginal**											
S: Cervical	NA	2.3	2.3	6.4^b^	7.6	.17	10.1^b^	NA	5.7	2.6	.93
S: Vaginal	NA	NA	NA	53.4	88.4	.006 (+)	NA	33.9^b^	32.1^b^	23.7^b^	.93
S: Vulvar	NA	6.9	4.2	25.5^b^	38.9	.01 (+)	22.5^b^	24.1^b^	10.8^b^	11.5^b^	.26
S: OPC	NA	NA	NA	NA	NA	NA	NA	NA	NA	NA	NA
S: Anal	66.7^b^	NA	NA	NA	NA	NA	NA	NA	9.8	NA	.97
**I: Vulvar**											
S: Cervical	4.1	2.0	1.6	3.4^b^	NA	.48	5.5^b^	2.6	2.4	7.8^b^	.03 (−)
S: Vaginal	NA	4.2	9.3^b^	29.3^b^	32.9^b^	.003 (+)	7.9	31.6^b^	18.8^b^	7.8^b^	.07
S: Vulvar	NA	7.2^b^	11.6^b^	57.2^b^	88.7^b^	<.001 (+)	4.7	58.5^b^	52.5^b^	24.5^b^	.01 (−)
S: OPC	4.0	3.4	3.9^b^	2.4	4.0^b^	.89	3.1	3.7^b^	3.5^b^	2.9^b^	.70
S: Anal	51.0^b^	3.1	7.2^b^	17.9^b^	13.7^b^	.48	4.5	12.1^b^	13.2^b^	16.6^b^	.24
**I: OPC**											
S: Cervical	2.1	1.4	1.6	1.1	2.2	.54	2.5	2.1^b^	0.4	1.3	.21
S: Vaginal	NA	2.6	3.9	NA	3.1	.82	4.3	3.0	NA	1.9	.49
S: Vulvar	4.5	1.0	1.7	3.4^b^	0.8	.93	1.4	3.3^b^	1.3	1.6	.41
S: OPC	32.5^b^	40.6^b^	49.5^b^	67.1^b^	81.4^b^	<.001 (+)	44.5^b^	55.3^b^	72.6^b^	53.8^b^	.45
S: Anal	NA	NA	2.2	0.8	NA	.64	NA	0.8	1.1	1.0	.68
**I: Anal**											
S: Cervical	NA	NA	1.8	1.5	1.2	.45	1.9	1.2	1.8	NA	.36
S: Vaginal	NA	NA	10.5^b^	13.6^b^	5.0	.61	NA	7.3	9.3^b^	11.4^b^	.40
S: Vulvar	NA	3.0	10.5^b^	10.0^b^	11.7^b^	.20	19.3^b^	11.1^b^	13.8^b^	2.1	<.001 (−)
S: OPC	NA	1.4	1.8	2.6	2.5	.48	NA	3.2^b^	0.8	2.8	.79
S: Anal	NA	NA	2.9	17.3^b^	23.2^b^	.004 (+)	17.1^b^	24.8^b^	8.5^b^	7.5^b^	.03 (−)

Abbreviations: HPV-SPC, human papillomavirus–associated second primary cancer; I, index cancer; NA, not applicable; OPC, oropharyngeal cancer; S, second primary cancer; SIR, standard incidence ratio.

^a^ (+) Denotes an increasing trend and (−) denotes a decreasing trend across the follow-up interval.

^b^ Statistically significant at *P* < .05.

**Table 4.** Standard Incidence Ratio of HPV-SPCs According to Year of HPV-SPC Diagnosis and Latency Interval for Men

Cancer Site	SIR by Year of HPV-SPC Diagnosis					*P* Value for Trend (Direction of Trend)^a^	SIR by Latency Interval, y				*P* Value for Trend (Direction of Trend)^a^
	1973-1979	1980-1989	1990-1999	2000-2009	2010-2014		<1	1-4	5-9	≥10	
**I: OPC**											
S: OPC	19.7^b^	21.6^b^	24.5^b^	21.3^b^	18.6	.06	15.0^b^	18.6^b^	26.1^b^	23.5^b^	<.001 (+)
S: Anal	NA	1.8	3.2	2.2	0.6	.43	1.5	2.0	2.3	0.9	.59
S: Penile	5.1	1.6	1.3	1.7	1.0	.46	4.0	1.4	2.2	NA	.22
**I: Anal**											
S: OPC	NA	5.2^b^	5.0^b^	1.6	1.1	.06	5.3^b^	3.2^b^	1.4	1.3	.12
S: Anal	NA	NA	26.5^b^	34.2^b^	74.0^b^	.009 (+)	14.5	58.1^b^	11.6^b^	64.7^b^	.47
S: Penile	NA	NA	NA	NA	8.2	NA	24.1	NA	NA	NA	NA
**I: Penile**											
S: OPC	3.5	1.7	4.7^b^	1.8	0.9	.35	3.5	2.6	2.8	1.7	.45
S: Anal	NA	NA	NA	4.3	NA	.66	NA	NA	6.2	NA	.99
S: Penile	NA	7.3	12.0^b^	59.3^b^	180.6^b^	<.001 (+)	15.2	79.0^b^	37.8^b^	48.1^b^	.56

Abbreviations: HPV-SPC, human papillomavirus–associated second primary cancer; I, index cancer; NA, not applicable; OPC, oropharyngeal cancer; S, second primary cancer; SIR, standard incidence ratio.

^a^ (+) Denotes an increasing trend across the follow-up interval.

^b^ Statistically significant at *P* < .05.

## Discussion

The results of our study show that survivors of HPV-associated cancers are at an increased risk of developing HPV-SPCs, many of which may be preventable. Among both women and men, the risk of developing HPC-SPC at the index cancer site was higher than that of developing HPC-SPC at a non–index cancer site; however, our subsite-specific analysis showed that among women with index vaginal, vulvar, and anal cancers the risk for all HPV-SPCs remained high. In contrast, the HPV-SPC risk was lowest among women with index cervical cancer. Among men, the oropharyngeal SPC risk was elevated after index penile and anal cancers. We found a persistently elevated risk of SPCs across latency intervals, which suggests that surveillance bias is an unlikely reason for elevated risk. Given the large difference in the HPV-SPC risk between survivors of HPV-associated cancers and that of non–HPV-associated cancers, it is likely that persistent HPV infection contributes to the development of HPV-SPCs among survivors of HPV-associated cancers.

Previous studies found a high risk of anal cancer among women with gynecological cancers,^28^ associations between anogenital and oropharyngeal cancers,^29^ and an increased risk of SPCs among cervical cancer survivors.^30,31,32,33^ Our study confirms these findings and adds to a growing body of evidence^34^ supporting a role of HPV in the development of SPCs. To our knowledge, our study is the first to show that the risk for most types of HPV-SPCs remains high after index vaginal and vulvar cancers among women, after index penile cancer among men, and after index anal cancer among women and men. This highlights the need to investigate the efficacy of secondary and tertiary prevention methods, which may include, for example, (1) screening for prevention (using cytology)^35^ or early detection (using digital anorectal examination)^36,37^ of anal cancer among women with gynecological precancers or cancers^38^ and (2) the role of adjuvant^39^ or therapeutic^40^ HPV vaccination for prevention of SPCs (by decreasing the risk of index HPV-associated cancers).^39,41,42,43^

Both women and men had an elevated risk of oropharyngeal SPC after any index cancer. Population-level screening for index oropharyngeal cancers is not feasible owing to our current inability to detect precancerous lesions. Furthermore, despite the high specificity (92%; 95% CI, 82%-97%) and moderate sensitivity (72%; 95% CI, 45%-89%) of oral HPV detection methods (oral rinses and oral swabs) for classifying HPV-positive oropharyngeal cancers,^44^ using these assays to screen healthy populations for index oropharyngeal cancers may not be efficient because oropharyngeal cancer is rare in the general population.^44^ However, using HPV detection methods to screen individuals who have already been treated for an HPV-associated cancer for oropharyngeal SPC might be an important public health opportunity. Another prevention method worth investigating in the secondary screening setting is ultrasound imaging,^45,46,47,48,49^ which has been shown to be able to identify cervical metastases in patients with early-stage oropharynx cancers.

Survivors of cervical cancer had the lowest HPV-SPC risk. One reason for this may be that the widespread implementation of cervical cancer screening and improvements in cervical cancer diagnosis, by enabling the detection and adequate management of precancerous lesions, provided greater protection against subsequent HPV-SPCs. We also found a decreasing trend in the risk of vulvar and oropharyngeal SPCs after index cervical cancer. A hypothesis for this could be that the prevention of index cervical cancer through the detection and management of cervical intraepithelial neoplasia might eradicate cervical HPV infection, thereby preventing SPCs at other sites, given a strong association between HPV infection occurring at oral, anal, and genital sites.^50,51,52^ Future studies of HPV infection persistence occurring at all anatomic sites among survivors of HPV-associated cancers are crucial to understanding the role of HPV in SPC carcinogenesis. Future economic evaluations are also needed to determine the value of continued surveillance tailored to the HPV-SPC risk.

### Strengths and Limitations

The principal strengths of our study included its large sample size, almost complete follow-up data, and high-quality control of the SEER program.^23^ Limitations specific to the SEER registry include that SPC at the same site is difficult to differentiate from the recurrent disease; therefore, it is likely that a small percentage of recurrence in adjacent anatomic sites (eg, oropharynx and oral cavity) could theoretically be misclassified as SPC. Even in clinical practice, these concerns are inevitable. To address this limitation, we conducted rigorous sensitivity analyses by increasing the latency interval to 1 year in our estimation of the HPV-SPC risk. In an extreme case scenario, we also estimated the risk of developing any HPV-SPC different than the index HPV-associated cancers, yet the risk was significantly greater. Another limitation of our study is that our classification of HPV-associated cancers was based on histologic types and not the actual assessment of individual tumor for the presence of HPV DNA; therefore, there is a possibility of misclassification, particularly for anatomic sites that have lower rates of HPV detection in tumors, such as the vulva (68.8%) and penis (63.3%).^18^

## Conclusions

Human papillomavirus infection may increase the HPV-SPC risk among survivors of HPV-associated cancers. Our findings suggest that HPV-SPC rates after index cervical cancer—the only HPV-associated cancer that has secondary prevention and surveillance recommendations—have declined in the last 4 decades. Vigilant monitoring and screening tailored to the HPV-SPC risks posed by specific HPV-associated cancers are crucial to decrease the mortality and economic burden of survivors of HPV-associated cancers and improve their quality of life.

## Notes

Supplement. **eTable 1.** *International Classification of Diseases for Oncology,* Site Codes, Histology Codes, and Additional Restrictions Used to Define Human Papillomavirus–Associated Index and Second Primary Cancers (SPCs) **eTable 2.** Sensitivity Analysis Using a Follow-up Period Beginning 1 Year After the Index Cancer Diagnosis **eTable 3.** Standard Incidence Ratios (SIRs) of Human Papillomavirus (HPV)-Related Second Primary Cancers After Index HPV-Related Cancers, Stratified by Gender, Index Cancer, and Second Primary Cancer **eFigure 1.** CONSORT Diagram Depicting Sample Flow of the Study Population **eFigure 2.** Cumulative Incidence of HPV-Associated Second Primary Cancers Among Women Diagnosed With Index HPV-Associated Index Primary Cancers **eFigure 3.** Cumulative Incidence of HPV-Associated Second Primary Cancers Among Men Diagnosed With Index HPV-Associated Index Primary Cancers **eReferences.**
