# Supplementary material for: Trends in Risks for Second Primary Cancers Associated With Index Human Papillomavirus–Associated Cancers
Source: JAMA Netw Open. 2018 Sep 7;1(5):e181999. doi: 10.1001/jamanetworkopen.2018.1999 (PMC6324459; doi:10.1001/jamanetworkopen.2018.1999)
Supplement: Supplement. — eTable 1. International Classification of Diseases for Oncology, Site Codes, Histology Codes, and Additional Restrictions Used to Define Human Papillomavirus–Associated Index and Second Primary Cancers (SPCs) eTable 2. Sensitivity Analysis Using a Follow-up Period Beginning 1 Year After the Index Cancer Diagnosis eTable 3. Standard Incidence Ratios (SIRs) of Human Papillomavirus (HPV)-Related Second Primary Cancers After Index HPV-Related Cancers, Stratified by Gender, Index Cancer, and Second Primary Cancer eFigure 1. CONSORT Diagram Depicting Sample Flow of the Study Population eFigure 2. Cumulative Incidence of HPV-Associated Second Primary Cancers Among Women Diagnosed With Index HPV-Associated Index Primary Cancers eFigure 3. Cumulative Incidence of HPV-Associated Second Primary Cancers Among Men Diagnosed With Index HPV-Associated Index Primary Cancers eReferences. [file jamanetwopen-1-e181999-s001.pdf]

Supplementary Online Content

Suk R, Mahale P, Sonawane K, et al. Trends in risks for second primary cancers associated with index human papillomavirus–associated cancers. *JAMA Netw Open*. 2018;1(5):e181999. doi:10.1001/jamanetworkopen.2018.1999

eTable 1. *International Classification of Diseases for Oncology* Site Codes, Histology Codes, and Additional Restrictions Used to Define Human Papillomavirus–Associated Index and Second Primary Cancers (SPCs)

eTable 2. Sensitivity Analysis Using a Follow-up Period Beginning 1 Year After the Index Cancer Diagnosis

eTable 3. Standard Incidence Ratios (SIRs) of Human Papillomavirus (HPV)-Related Second Primary Cancers After Index HPV-Related Cancers, Stratified by Gender, Index Cancer, and Second Primary Cancer

eFigure 1. CONSORT Diagram Depicting Sample Flow of the Study Population

eFigure 2. Cumulative Incidence of HPV-Associated Second Primary Cancers Among Women Diagnosed With Index HPV-Associated Index Primary Cancers

eFigure 3. Cumulative Incidence of HPV-Associated Second Primary Cancers Among Men Diagnosed With Index HPV-Associated Index Primary Cancers

eReferences.

This supplementary material has been provided by the authors to give readers additional information about their work.

© 2018 Suk R et al. *JAMA Network Open*.

eTable 1. International Classification of Diseases for Oncology Site Codes, Histology Codes, and Additional Restrictions Used to Define Human Papillomavirus–Associated Index and Second Primary Cancers (SPCs)

Index and SPC Sites [1-5]	ICD-O-3 Site Codes	ICD-O-3 Histology Codes	Additional Restrictions
Squamous cell carcinoma of the oropharynx	C01.9, 02.4, 02.8, 05.1–05.2, 09.0–09.1, 09.8–09.9, 10.0–10.9, 10.8–10.9, 14.0, 14.2, 14.8	8050–8084, 8120–8131	Restrict to microscopically confirmed
Squamous cell carcinoma of the anus	C20.9	8050–8084, 8120–8131	Restrict to microscopically confirmed
Squamous cell carcinoma of the vulva	C51.0–51.9	8050–8084, 8120–8131	Restrict to microscopically confirmed
Squamous cell carcinoma of the vagina	C52.9	8050–8084, 8120–8131	Restrict to microscopically confirmed
Carcinoma of the cervix	ICD-O-3 Site Codes: C53.0–53.9	8010–8671, 8940–8941	Restrict to microscopically confirmed
Squamous cell carcinoma of the penis	C60.0–60.9	8050–8084, 8120–8131	Restrict to microscopically confirmed

Abbreviation: ICD-O-3, International Classification of Diseases for Oncology, 3rd Edition

© 2018 Suk R et al. *JAMA Network Open*.

eTable 2. Sensitivity Analysis Using a Follow-up Period Beginning 1 Year After the Index Cancer Diagnosis

Index HPV-Associated Cancer	Women						Men					
	All HPV-SPC			All HPV-SPC (same site excluded)			All HPV-SPC			All HPV-SPC (same site excluded)		
	Observed	SIR (95% CI)	EAR*	Observed	SIR (95% CI)	EAR*	Observed	SIR (95% CI)	EAR*	Observed	SIR (95% CI)	EAR*
**All HPV-related cancers**	1,278	6.2 (5.9-6.6)	18.1	443	3.5 (3.1-3.8)	5.3	1,098	15.8 (14.9-16.8)	53.5	35	1.9 (1.3-2.6)	1.0
**Cervical**	333	2.4 (2.1-2.6)	4.3	272	3.5 (3.1-3.9)	4.4	NA	NA	NA	NA	NA	NA
**Vaginal**	22	5.9 (3.7-9.0)	23.0	15	4.3 (2.4-7.1)	14.5	NA	NA	NA	NA	NA	NA
**Vulvar**	255	13.6 (12.0-15.3)	54.4	82	5.5 (4.4-6.8)	15.5	NA	NA	NA	NA	NA	NA
**Oropharyngeal**	602	20.5 (18.9-22.2)	83.8	31	1.6 (1.1-2.2)	1.6	904	18.8 (17.6-20.1)	65.1	12	1.6 (0.8-2.8)	0.3
**Anal**	66	5.1 (3.9-6.4)	17.2	43	3.8 (2.7-5.1)	10.3	37	6.4 (4.5-8.8)	18.5	10	1.9 (0.9-3.5)	2.8
**Penile**	NA	NA	NA	NA	NA	NA	46	7.3 (5.4-9.7)	24.0	13	2.3 (1.2-3.9)	4.4

Abbreviations: HPV, human papillomavirus; HPV-SPC**,** HPV-related second primary cancer; SIR, standard incidence ratio; CI, confidence interval; EAR, excess absolute risk.

[a] EAR = (observed count – expected count) * 10,000 / person-years at risk.

© 2018 Suk R et al. *JAMA Network Open*.

eTable 3. Standard Incidence Ratios (SIRs) of Human Papillomavirus (HPV)-Related Second Primary Cancers After Index HPV-Related Cancers, Stratified by Gender, Index Cancer, and Second Primary Cancer

Cancer sites	Women					Men		
	**I: Cervical (42,273 P 473,820 PY)**	**I: Vaginal (1,543 P 9,057 PY)**	**I: Vulvar (6,642 P 48,373 PY)**	**I: Oropharyngeal (14,453 P 78,847 PY)**	**I: Anal (5,038 P 34,594 PY)**	**I: Penile (2,597 P 18,520 PY)**	**I: Oropharyngeal (32,171 P 154,757 PY)**	**I: Anal (3,086 P 19,163 PY)**
**S: Cervical**								
**Observed**	72	5	14	17	5	NA	NA	NA
**Expected**	68.3	1.5	6.5	10.9	4.4			
**SIR (95% CI)**	1.1 (0.8-1.3)	3.4 (1.1-7.9)	2.2 (1.2-3.6)	1.6 (0.9-2.5)	1.1 (0.4-2.6)			
**S: Vaginal**								
**Observed**	121	7	23	4	7	NA	NA	NA
**Expected**	7.0	0.3	1.2	1.9	0.8			
**SIR (95% CI)**	17.3 (14.3-20.6)	26.1 (10.5-53.8)	18.5 (11.8-27.8)	2.1 (0.6-5.4)	8.4 (3.4-17.3)			
**S: Vulvar**								
**Observed**	79	13	175	13	27	NA	NA	NA
**Expected**	20.9	00.8	4.3	6.3	2.8			
**SIR (95% CI)**	3.8 (3.0-4.7)	16.6 (8.8-28.4)	40.6 (34.8-47.1)	2.1 (1.1-3.5)	9.6 (6.3-13.9)			
**S: Oropharyngeal**								
**Observed**	56	0	22	632	10	14	992	13
**Expected**	39.6	1.3	6.6	10.9	4.7	5.7	47.2	5.4
**SIR (95% CI)**	1.4 (1.1-1.8)	NA	3.3 (2.1-5.1)	57.9 (53.5-62.6)	2.1 (1.0-3.9)	2.5 (1.3-4.1)	21.0 (19.7-22.4)	2.4 (1.3-4.1)
**S: Anal**								
**Observed**	34	1	31	3	26	1	9	28
**Expected**	15.1	0.4	2.4	3.7	1.8	0.7	5.1	0.7
**SIR (95% CI)**	2.3 (1.6-3.2)	2.3 (0.1-12.8)	13.2 (8.9-18.7)	0.8 (0.2-2.4)	14.6 (9.5-21.3)	1.5 (0.04-8.4)	1.8 (0.8-3.4)	43.0 (28.6-62.2)
**S: Penile**								
**Observed**	NA	NA	NA	NA	NA	34	6	1
**Expected**						0.7	3.7	0.4
**SIR (95% CI)**						52.5 (36.4-73.4)	1.6 (0.6-3.5)	2.3 (0.1-12.9)

Abbreviations: I, Index; S, second primary; P, persons; PY, person years; SIR, standard incidence ratio; CI, confidence intervals; NA, not applicable

© 2018 Suk R et al. *JAMA Network Open*.

eFigure 1. CONSORT Diagram Depicting Sample Flow of the Study Population

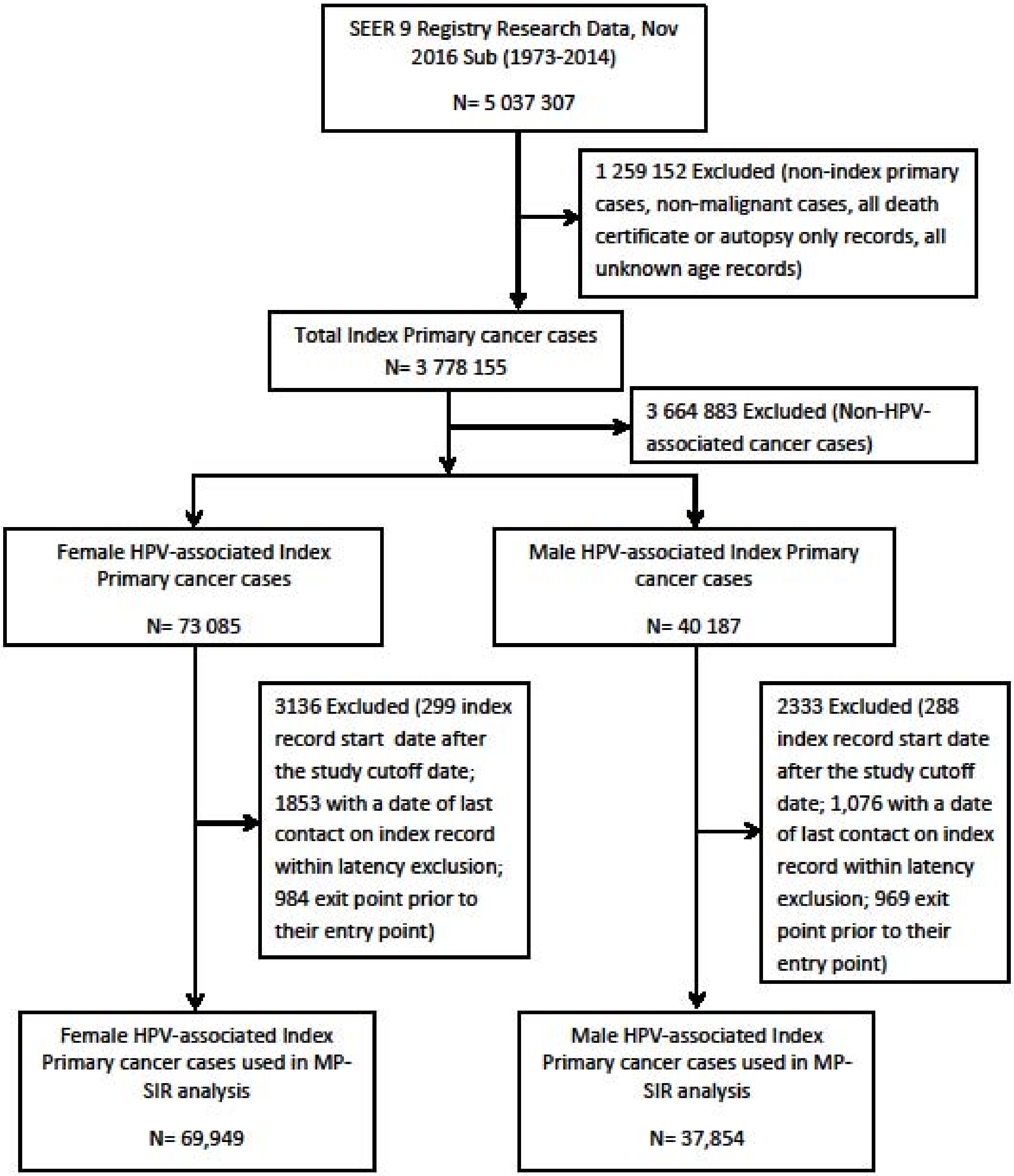

© 2018 Suk R et al. *JAMA Network Open*.

eFigure 2. Cumulative Incidence of HPV-Associated Second Primary Cancers Among Women Diagnosed With Index HPV-Associated Index Primary Cancers

A

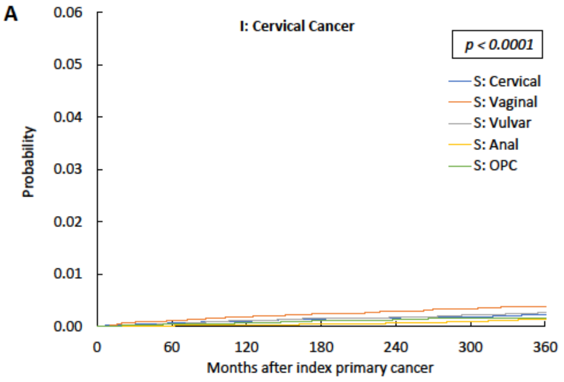

No. at risk	44 011	25 414	19 549	14 801	10 307	6619	3739
Cumulative counts of SPCs by subsite							
Cervical	0	10	21	39	52	60	66
Vaginal	0	22	48	66	88	101	113
Vulvar	0	11	23	41	49	60	67
Anal	0	3	7	10	13	18	26
OPC	0	9	17	29	35	43	49

B

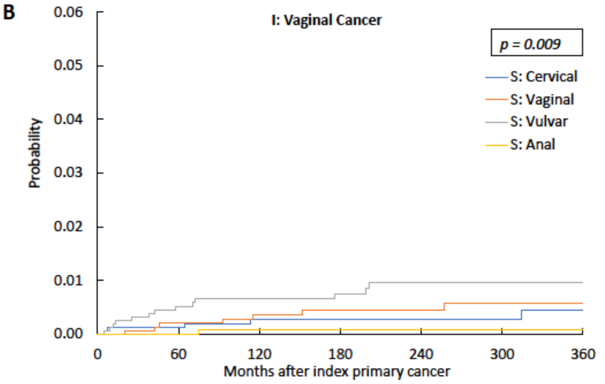

No. at risk	1673	601	366	221	122	49	24
Cumulative counts of SPCs by subsite							
Cervical	0	1	2	3	3	4	5
Vaginal	0	3	4	6	6	7	7
Vulvar	0	3	8	10	12	12	13
Anal	0	1	1	1	1	1	1
OPC	N/A	N/A	N/A	N/A	N/A	N/A	N/A

C

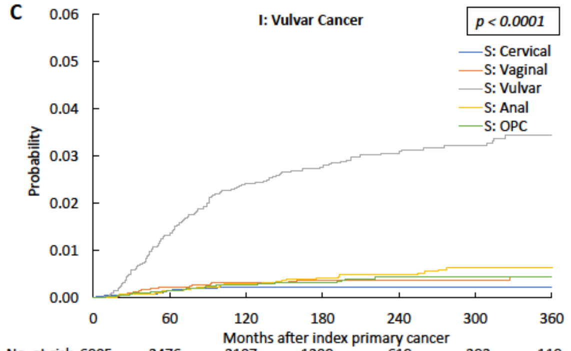

No. at risk	6905	3476	2107	1209	619	293	119
Cumulative counts of SPCs by subsite							
Cervical	0	3	10	13	14	14	14
Vaginal	0	8	17	21	22	22	23
Vulvar	0	38	101	139	158	167	173
Anal	0	5	12	17	22	26	26
OPC	0	5	9	16	19	22	22

D

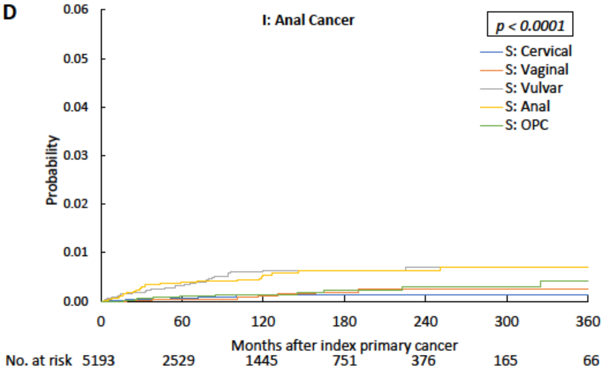

No. at risk	5193	2529	1445	751	376	165	66
Cumulative counts of SPCs by subsite							
Cervical	0	3	4	5	5	5	5
Vaginal	0	2	3	5	6	7	7
Vulvar	0	9	20	24	25	25	26
Anal	0	9	17	21	24	25	25
OPC	0	3	4	5	7	9	10

E

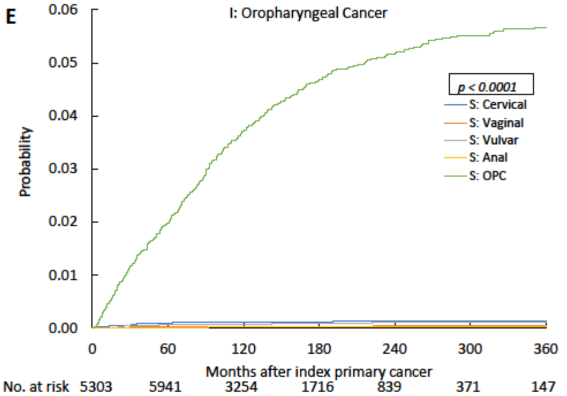

No. at risk	5303	5941	3254	1716	839	371	147
Cumulative counts of SPCs by subsite							
Cervical	0	9	11	14	15	16	16
Vaginal	0	2	2	3	4	4	4
Vulvar	0	3	5	7	10	13	13
Anal	0	0	2	3	3	3	3
OPC	0	148	324	469	545	601	617

© 2018 Suk R et al. *JAMA Network Open*.

Figure illustrates subsite-specific cumulative incidence curves developing of HPV-associated second primary cancers among women diagnosed with index HPV-associated cancers. **Panel A** illustrates cumulative incidence of developing second primary cervical, vaginal, vulvar, anal, and oropharyngeal cancers after index cervical cancer. **Panel B** illustrates cumulative incidence of developing second primary cervical, vaginal, vulvar, and anal cancers after index vaginal cancer. **Panel C** illustrates cumulative incidence of developing second primary cervical, vaginal, vulvar, anal, and oropharyngeal cancers after index vulvar cancer. **Panel D** illustrates cumulative incidence of developing second primary cervical, vaginal, vulvar, anal, and oropharyngeal cancers after index anal cancer. **Panel E** illustrates cumulative incidence of developing second primary cervical, vaginal, vulvar, anal, and oropharyngeal cancers after index oropharyngeal cancer.

I = Index cancer; S = Second primary cancer; OPC = oropharyngeal cancer

© 2018 Suk R et al. *JAMA Network Open*.

eFigure 3. Cumulative Incidence of HPV-Associated Second Primary Cancers Among Men Diagnosed With Index HPV-Associated Index Primary Cancers

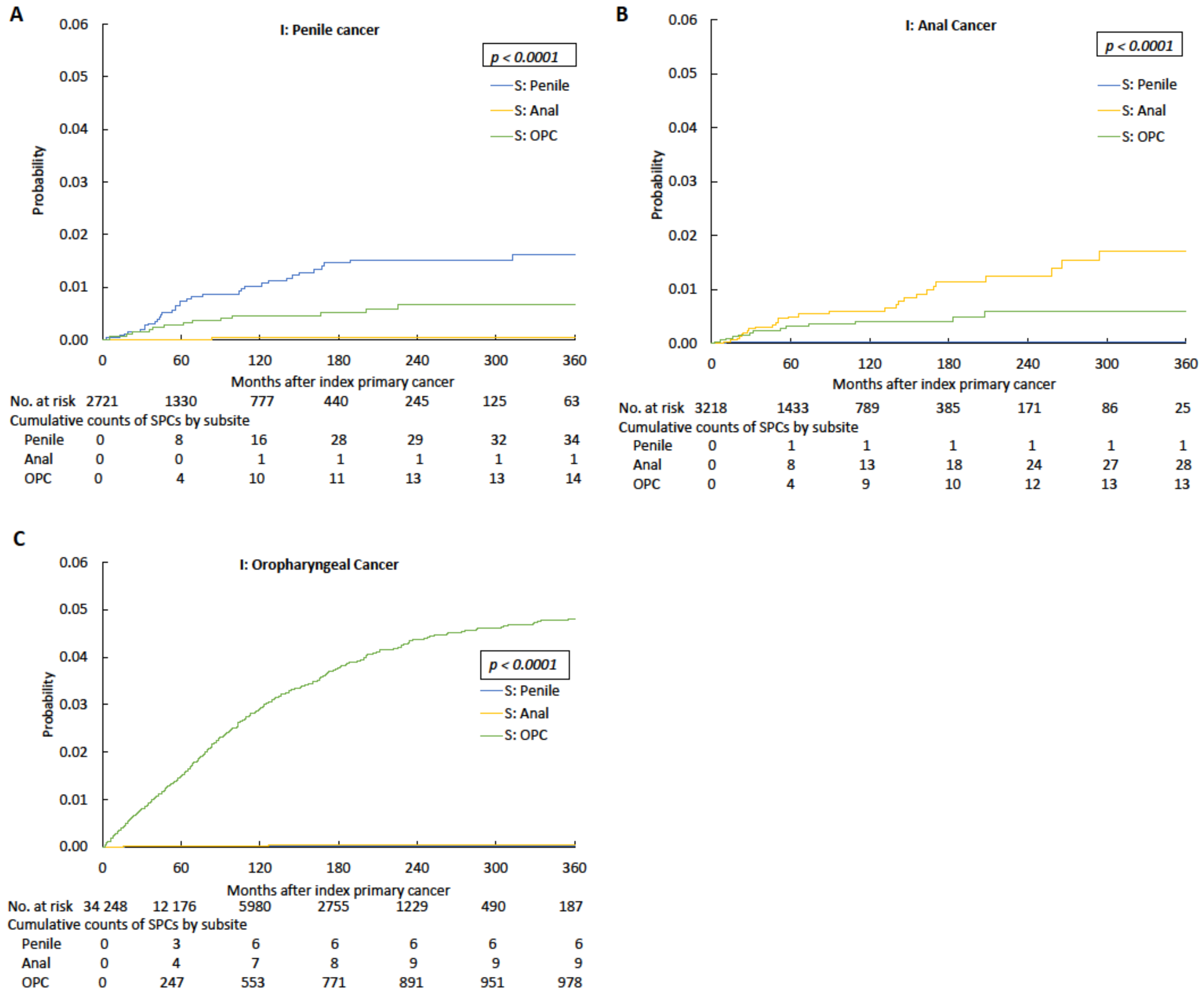

© 2018 Suk R et al. *JAMA Network Open*.

Figure illustrates subsite-specific cumulative incidence curves developing of HPV-associated second primary cancers among men diagnosed with index HPV-associated cancers. **Panel A** illustrates cumulative incidence of developing second primary penile, anal, and oropharyngeal cancers after index penile cancer. **Panel B** illustrates cumulative incidence of developing second primary penile, anal, and oropharyngeal cancers after index anal cancer. **Panel C** illustrates cumulative incidence of developing second primary penile, anal, and oropharyngeal cancers after index oropharyngeal cancer.

© 2018 Suk R et al. *JAMA Network Open*.

eReferences

1. Viens LJ, Henley SJ, Watson M, et al. Human Papillomavirus-Associated Cancers - United States, 2008-2012. *MMWR Morb Mortal Wkly Rep.* Jul 8 2016;65(26):661-666.

2. Saraiya M, Unger ER, Thompson TD, et al. US assessment of HPV types in cancers: implications for current and 9-valent HPV vaccines. *J Natl Cancer Inst.* Jun 2015;107(6):djv086.

3. Watson M, Saraiya M, Ahmed F, et al. Using population-based cancer registry data to assess the burden of human papillomavirus-associated cancers in the United States: overview of methods. *Cancer.* Nov 15 2008;113(10 Suppl):2841-2854.

4. International Agency for Research on Cancer. *IARC monographs on the evaluation of carcinogenic risks to humans.* Lyon, France: International Agency for Research on Cancer; 20072007.

5. Centers for Disease Control and Prevention. *How Many Cancers Are Linked with HPV Each Year?* Atlanta, GA: U.S.: Department of Health and Human Services;2017.

© 2018 Suk R et al. *JAMA Network Open*.
